# 2-Chloroethanol Induced Upregulation of Matrix Metalloproteinase-2 in Primary Cultured Rat Astrocytes Via MAPK Signal Pathways

**DOI:** 10.3389/fnins.2016.00593

**Published:** 2017-01-04

**Authors:** Qi Sun, Yingjun Liao, Tong Wang, Hongge Tang, Gaoyang Wang, Fenghong Zhao, Yaping Jin

**Affiliations:** ^1^Department of Occupational and Environmental Health, School of Public Health, China Medical UniversityShenyang, China; ^2^Department of Physiology, China Medical UniversityShenyang, China

**Keywords:** 1,2-dichloroethane, astrocytes, 2-chloroethanol, matrix metalloproteinase-2, mitogen-activated protein kinase signal pathways

## Abstract

This study was to explore the mechanisms underlying 1,2-dichloroethane (1,2-DCE) induced brain edema by focusing on alteration of matrix metalloproteinase-2 (MMP-2) in rat astrocytes induced by 2-chloroethanol (2-CE), an intermediate metabolite of 1,2-DCE *in vivo*. Protein and mRNA levels of MMP-2, and the phosphorylated protein levels of p38 MAPK (p-p38), extracellular signal regulated protein kinase (p-ERK1/2) and c-Jun N-terminal kinase (p-JNK1/2) in astrocytes were examined by immunostaining, western blot or real-time RT-PCR analysis. Findings from this study disclosed that protein levels of MMP-2 were upregulated by 2-CE in astrocytes. Meanwhile, protein levels of p-p38, p-ERK1/2 and p-JNK1/2 were also increased apparently in the cells treated with 2-CE. Moreover, pretreatment of astrocytes with SB202190 (inhibitor of p38 MAPK), U0126 (inhibitor of ERK1/2) or SP600125 (inhibitor of JNK1/2) could suppress the upregulated expression of p-p38, p-ERK1/2, and p-JNK1/2. In response to suppressed protein levels of p-p38 and p-JNK1/2, the protein levels of MMP-2 also decreased significantly, indicating that activation of MAPK signal pathways were involved in the mechanisms underlying 2-CE-induced upregulation of MMP-2 expression.

## Introduction

The compound of 1,2-dichloroethane (1,2-DCE, CAS number: 107-06-2) is one of the most widely produced halocarbons, and used mainly in the production of vinyl chloride worldwide. This chemical is also used as the general organic solvent, especially as the thinner of adhesives in some countries. It is clear that subacute poisoning of 1,2-DCE can cause toxic encephalopathy in the exposed workers. The postmortem and clinical studies showed that brain edema is the main pathological change and the cause of death in the poisoned workers (Liu et al., 2010; Zhang et al., 2011; Chen et al., 2015). But so far, little is known about the molecular mechanisms underlying 1,2-DCE-induced brain edema.

Accumulated evidences have suggested that the principal pathway involving metabolism of 1,2-DCE is mediated by microsomal cytochrome P450 2E1 (CYP2E1) in animals and most probably, in humans as well (Reitz et al., 1982; Sweeney et al., 2008). The metabolites generated in CYP2E1 mediated metabolism of 1,2-DCE are 2-chloroethanol (2-CE), chloroacetaldehyde and chloroacetic acid. Of which, 2-CE and chloroacetaldehyde are the reactive intermediates, and assumed to be involved in mechanisms underlying 1,2-DCE induced brain edema since they are more reactive than their parent compound (Guengerich et al., 1980; Igwe et al., 1986; Raucy et al., 1993). Hence, it is essential for exploring the neurotoxic effects induced directly by 2-CE.

Brain edema has been classically defined as cytotoxic and vasogenic edema referred to either intracellular or extracellular water excess. The former is due to energy failure in injured brain cells, while the latter results from breakdown in blood-brain barrier (BBB) (Simard and Rivest, 2007). Recently, roles of matrix metalloproteinases (MMPs) in the pathogenesis of vasogenic brain edema are actively investigated. Deregulation of MMPs, the largest class of human proteases, has been implicated in brain damage in both animal and human studies (Morancho et al., 2010; Iwado et al., 2012; Yan et al., 2016).

MMPs are a gene family of zinc-dependent proteases involved in the regulation of extracellular matrix composition (Sternlicht and Werb, 2001). As the main gelatinases in the brain, MMP-2 and MMP-9 can degrade collagen IV and fibronectin in microvascular basal lamina around the endothelial cells. The tight junction proteins, occludin and claudin-5 in the endothelial barrier, are also vulnerable to attack by MMPs. Thereby excessive expression of MMP-2 and MMP-9 can disrupt the BBB integrity, which might contribute to development of vasogenic brain edema (Asahi et al., 2001a,b; Planas et al., 2001; Yang et al., 2007; Higashida et al., 2011; Ramos-Fernandez et al., 2011; Suofu et al., 2012). It has been reported that in most cases, the inhibitory effects on expressions of MMP-2 and MMP-9 could reduce the permeability of BBB at the early phase of brain edema formation (Romanic et al., 1998; Rosenberg et al., 1998; Gasche et al., 1999).

Although MMP-2 and MMP-9 have similar substrate specificities, the regulation ways in their expression are quite different. A variety of studies have disclosed that MMP-2 is constitutively expressed, and normally present in an inactive form tethered to the cell surface; however, MMP-9 is normally expressed at low levels but is markedly up-regulated in many brain diseases (Senior et al., 1991; Okada et al., 1992; Nag et al., 2009). Our recent study has demonstrated that levels of MMP-2 protein but not its mRNA, whereas levels of MMP-9 mRNA were increased apparently in the cerebral tissues of mice during the brain edema formation induced by subacute poisoning of 1,2-DCE (Wang et al., 2014). However, up to date, little is known about the cellular source of these MMPs or how their expression is regulated in the brain of 1,2-DCE poisoned mice. Accordingly, the changes of MMP-2 and MMP-9 expression, and the roles of mitogen-activated protein kinase (MAPK) signal pathways in their regulation were explored in 2-CE treated rat astrocytes. In this paper, the changes of MMP-2 expression, and the involvement of MAPK signal pathways in its regulation in 2-CE treated rat astrocytes were reported.

Astrocytes, the most numerous cell type in the brain, play essential roles in regulation of homeostatic balance and maintenance of BBB integrity (Chambaut-Guérin et al., 2000; Ransom and Ransom, 2012; Finsterwald et al., 2015). The previous studies have disclosed that MMP-2 is expressed in several cell types in the brain, such as astrocytes, endothelial cells and microglia (Ogier et al., 2006; Qin et al., 2015; Maeda et al., 2016). The *in vitro* studies demonstrated that MMP-2 in rat astrocytes could be induced by the proinflammatory cytokine, such as IL-1β and TNF-α (Gottschall and Deb, 1996; Ogier et al., 2006; Wang et al., 2006). In addition, it has been reported that MMP-2 was upregulated in response to transient focal cerebral ischemia in the early stages of the injury, and then degraded the proteins in the tight junctions of BBB, thus leading to breakdown in BBB integrity (Chang et al., 2003; Yang et al., 2007).

The MAPK signal pathways transmit signals from cell membrane to nucleus in response to a variety of stimuli. Because these pathways control a broad spectrum of cellular processes, they are accepted as the important regulators in pathogenesis of human diseases. The most extensively studied subfamilies of MAPK signal pathways are extracellular signal-regulated kinase 1/2 (ERK1/2), p38 MAPK (p38), and c-Jun amino terminal kinase (JNK) (Arai et al., 2003; Tejima et al., 2007; Hsieh et al., 2010; Ralay-Ranaivo et al., 2011). They are activated through phosphorylation by upstream kinases recruited through diverse extracellular signal events. Upon activation of MAPK signal pathways, transcription factors that are present in the cytoplasm or nucleus are activated, leading to expression of a variety of target genes and resulting in a series of biological responses (Hsieh et al., 2004, 2006; Wu et al., 2004).

Taken together, it was of interest to investigate the involvement of MAPK signal pathways in modulation of MMP-2 expression in 2-CE treated astrocytes, which would contribute to identify new therapeutic targets for 1,2-DCE induced brain edema. To the best of our knowledge, no studies concerning this subject have been reported.

## Materials and methods

### Animal care and use statement

All animal studies were approved by the Scientific Research Committee of China Medical University and have been conducted in accordance with Chinese National Guidelines for the Care and Use of Laboratory animal in animal experiments.

### Reagents

2-CE with purity more than 99.0% was obtained from the Sinopharm Chemical Reagent Co., Ltd, China. Dulbecco's modified Eagle's medium (DMEM), trypsin, penicillin and streptomycin were purchased from Invitrogen (USA). Heat inactivated fetal bovine serum (FBS) was the product of Biological Industries (Israel). Trizol reagent was obtained from Takara (Japan). RIPA Lysis Buffer was purchased from Beyotime Biotechnology (China). The enhanced chemiluminescence (ECL) plus kit and bicinchoninic acid (BCA) protein assay kit were obtained from Pierce (Thermo Fisher Scientific, USA). The polyclonal antibody against MMP-2 was purchased from Millipore (USA, ^#^AB19167). The primary antibodies against phosphorylated p38 (p-p38, ^#^9211), phosphorylated ERK1/2 (p-ERK1/2, ^#^4370), phosphorylated JNK1/2 (p-JNK1/2, ^#^9251) and β-actin (^#^4970) were the products of Cell Signaling Technology (USA). SB202190, U0126, SP600125 were purchased from Selleck (USA). Secondary antibody conjugated fluorescein isothiocyanate (FITC), tetraethyl rhodamine isothiocyanate (TRITC) or horseradish peroxidase were purchased from the ZSGB Biotechnology (China). All other chemicals were of the analytical grade and obtained from the local chemical suppliers.

These chemical reagents were prepared as stock solutions with sterile water, and then diluted to the final concentrations before application. Water used in this study was doubly distilled.

### Primary culture for astrocyte

The cerebral cortices of postnatal day 1–3 Wistar rats, obtained from the animal laboratory of China Medical University were carefully removed, and washed three times by ice-cold Hanks balanced salt solution without Ca^2+^ and Mg^2+^. They were chopped into pieces less than 1 mm on each side, and then dissociated with 0.125% (w/v) trypsin solution at 37°C for 20 min with gentle shaking. The cell suspension was filtered through a 200 mesh (76 μm) stainless steel screen followed by centrifugation. Dissociated cells were suspended in DMEM containing 20% FBS and 1% penicillin-streptomycin. They were plated in the culture dishes pre-coated with poly-L-lysine at a density of 1 × 10^6^/ml, and maintained at 37°C, in 5% CO_2_ and 100% humidified atmosphere with replacement of fresh medium every 3 days.

**Figure 1:**
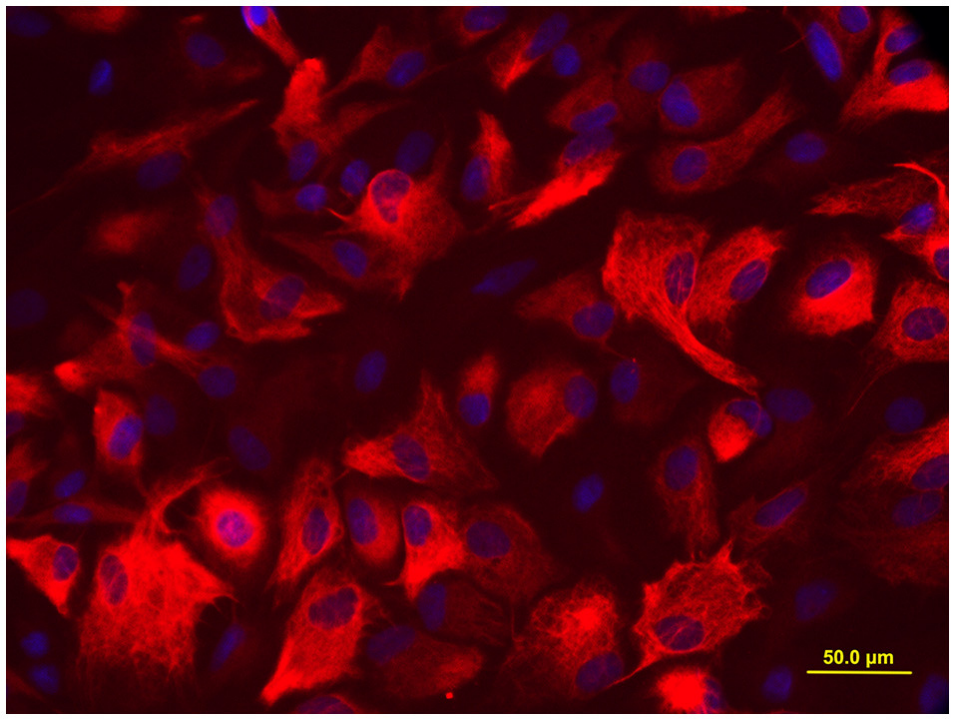
**Immunofluorescence staining for GFAP**. More than 95% cells were positive for astrocyte biomarker GFAP, the photomicrographs were captured with an Olympus fluorescence microscope (400 ×).

When a confluent layer was formed, the culture dishes were shaken for 15 h at 250 rpm in the orbital shaker to remove oligodendrocyte precursors and microglia. Following shaking, the medium was changed immediately, and a nearly pure layer of astrocyte was obtained in the culture dishes. The target cells were grown for 24 h, and then trypsinized and reseeded in new culture dishes at a density of 1 × 10^5^/ml. As illustrated in Figure 1, more than 95% cells were positive for glial fibrillary acidic protein (GFAP, a marker of astrocyte), determined by immunofluorescence staining.

### Treatments

The stock solution of 1 M (mol/L) 2-CE was prepared with redistilled water, and then diluted to the target concentrations by the media containing 5% FBS before application. The present study included two parts of experiments. For part one, astrocytes were divided randomly into four groups, the control group and three exposure groups. The cells in these groups were exposed to 0, 7.5, 15, or 30 mM 2-CE for 24 h in the media with 5% FBS when a confluent layer was formed. After exposure, the cells were collected for analysis of MMP-2 expression affected by 2-CE.

For part two, the cells were divided into seven groups to explore the involvement of MAPK signal pathways in regulation of MMP-2 expression in astrocytes affected by 2-CE. They were blank control, solvent control, and inhibitor control, exposure group, and low, middle and high dose intervention groups, respectively. Cells in the solvent control were treated with dimethyl sulfoxide (DMSO, the concentration in the media was less than 0.1%). Cells in the inhibitor control were treated with 30 μM SB202190, 10 μM U0126, or 10 μM SP600125, which were dissolved into DMSO, and then added into the media (Wu et al., 2004). Cells in the exposure group and intervention groups were exposed to 30 mM 2-CE for 24 h as described above. Moreover, cells in the intervention groups were pretreated 1 h before exposure to 2-CE with 1, 10, and 30 μM SB202190 for inhibition of p38 (Manthey et al., 1998), 0.1, 1.0, and 10.0 μM U0126 for inhibition of ERK1/2 (Smutny et al., 2014), or 0.1, 1.0, and 10.0 μM SP600125 for inhibition of JNK1/2 (Bennett et al., 2001).

After treatment, these cells were collected for analysis of protein and gene levels of MMP-2, and protein levels of p-p38, p-ERK1/2 and p-JNK1/2.

### Analysis

#### Immunocytochemical staining

Briefly, cells in the 12-well culture plate were fixed with 4% paraformaldehyde, permeabilized in 0.2% Triton X-100, and incubated with 10% normal goat serum at room temperature to block nonspecific binding of antiserum. They were then incubated with primary antibodies against GFAP (1:400, mouse anti-rat) or MMP-2 (1:100, rabbit anti-rat) at 4°C overnight. Labeled cells were visualized by TRITC conjugated goat anti-mouse or FITC conjugated goat anti-rabbit secondary antibody and observed under a fluorescence microscope (Olympus BX51). Digital images were captured using digital camera system (Olympus SC35). For negative controls, the primary antibodies were omitted. All steps were carried out in a humidified chamber.

#### Western blot analysis

At the end of exposure, the cells were detached by scraping, and harvested by centrifugation. Cell pellets were suspended and lysed by RIPA buffer. Protein concentrations in the lysates were measured with a BCA protein assay kit. Aliquots of each sample (40 μg per lane) were loaded to 10% SDS-PAGE, and then separated by electrophoresis. Blots were transferred onto polyvinylidene difluoride (PVDF) membranes (Millipore, USA), and subsequently probed with the primary antibodies against MMP-2 (1:1000, rabbit anti-rat), p-p38 (Thr^180^/Tyr^182^, 1:500, rabbit anti-rat), p-ERK1/2 (Thr^202^/Tyr^204^, 1:500, rabbit anti-rat), p-JNK1/2 (Thr^183^/Tyr^185^, 1:500, rabbit anti-rat), and β-actin (1:2000, rabbit anti-rat) at 4°C overnight. The bands were detected by the secondary antibodies conjugated with horseradish peroxidase (1:5000) for 1 h at room temperature, and visualized by using the ECL plus kit. The integrated intensity of the target protein was semi-quantitatively assessed by densitometry using an image analyzing software (Gel-Pro analyzer v4.0), and then normalized to β-actin levels from the same blot.

#### Levels of MMP-2 mRNA by quantitative real-time RT-PCR

The quantitative real-time RT-PCR was conducted according to the Minimum Information for Publication of Quantitative Real-Time PCR Experiments (MIQE) guidelines. Total RNA was isolated from the cells using Trizol Reagent. At first, the cDNA was synthesized from the total RNA by using the PrimeScript RT reagent kit (Takara, Japan). Thereafter, the cDNA was served as templates for real-time PCR amplification by using the SYBR® Premix Ex Taq II (Takara, Japan) and ABI 7500 Real-Time PCR System (Applied Biosystems, USA). Amplification was conducted for 40 cycles of 5 s at 95°C and 34 s at 60°C. Results were analyzed using the comparative Ct method as described by Livak and Schmittgen (2001). RNA abundance was expressed as 2^−ΔΔCt^ for the target gene normalized against the GAPDH gene (as the reference gene), and presented as fold change versus control sample. The following primer pairs were used: MMP-2: sense 5′-GCA ACC ACA ACC AAC TAC GA-3′; antisense 5′-CCA GTG TCA GTA TCA GCA TCA G-3′ (NM_031054.2, GI: 146262018). GAPDH: sense 5′-GCA AGA GAG AGG CCC TCA G-3′; antisense 5′-TGT GAG GGA GAT GCT CAG TG-3′ (NM_017008.4, GI: 402691727).

### Statistical analysis

Data were expressed as mean ± standard deviation (SD), and analyzed using the SPSS for Windows, version 20.0 (SPSS Inc., USA). Significant differences among group means were evaluated by analysis of variance test (one-way ANOVA). Post hoc tests were analyzed by Student-Newman-Keuls (SNK) test. The statistical significance was defined as *p* < 0.05.

## Results

### Expressions of MMP-2 in astrocytes affected by 2-CE treatment

**Figure 2:**
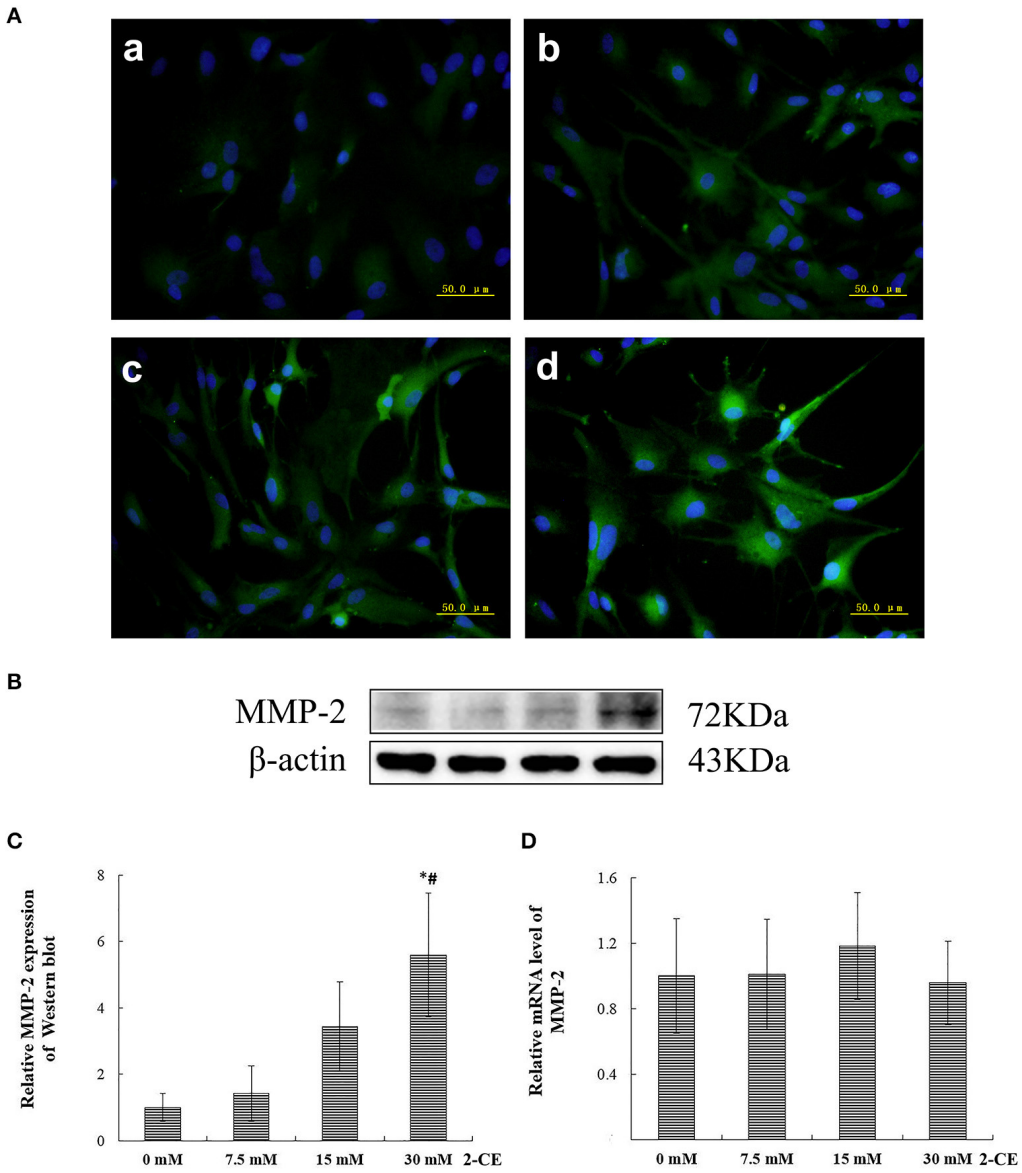
**Changes of MMP-2 protein and mRNA levels in primary cultured astrocytes exposed to 2-CE**. **(A)** Immunofluorescence staining for MMP-2 (400 ×). a: control; b: 7.5 mM dose; c: 15 mM dose; d: 30 mM dose 2-CE. **(B)** Western blot analysis. Images were the representative results of three separate experiments for each group. **(C)** Densitometric analysis of western blots for MMP-2. The relative intensity in arbitrary units compared to β-actin and presented as fold change vs the control group. **(D)** Quantitation of MMP-2 mRNA by real-time RT-PCR. The gene expression was normalized to GAPDH and presented as fold change vs. the control group. Data expressed as mean ± SD were the results of three independent experiments from different litters of rats for each treatment, and analyzed by One-way ANOVA. Significant difference was defined as *p* < 0.05, and ^*^, vs. control; ^#^, vs. 7.5 mM dose.

The representative micrographs in Figure 2A illustrated the immunoreactivities to MMP-2 in the cells from different groups. The fluorescence intensities in the exposure groups increased apparently 15 and 30 mM 2-CE exposure groups. Consistent with the results of immunostaining, protein levels of MMP-2 in 30 mM 2-CE exposure groups increased significantly as compared to the control and 7.5 mM 2-CE exposure groups (*p* < 0.05, shown in Figures 2B,C). In addition, the graph shown in Figure 2D disclosed the comparison of MMP-2 mRNA levels among groups, and indicated that there was not any significant difference (*p* < 0.05).

Accordingly, the protein expression of MMP-2 in rat astrocytes could be upregulated confirmatively by exposure to 30 mM 2-CE. Thus, this dosage was employed in the following studies.

### Involvement of p38 in upregulation of MMP-2 expression in 2-CE treated astrocytes

**Figure 3:**
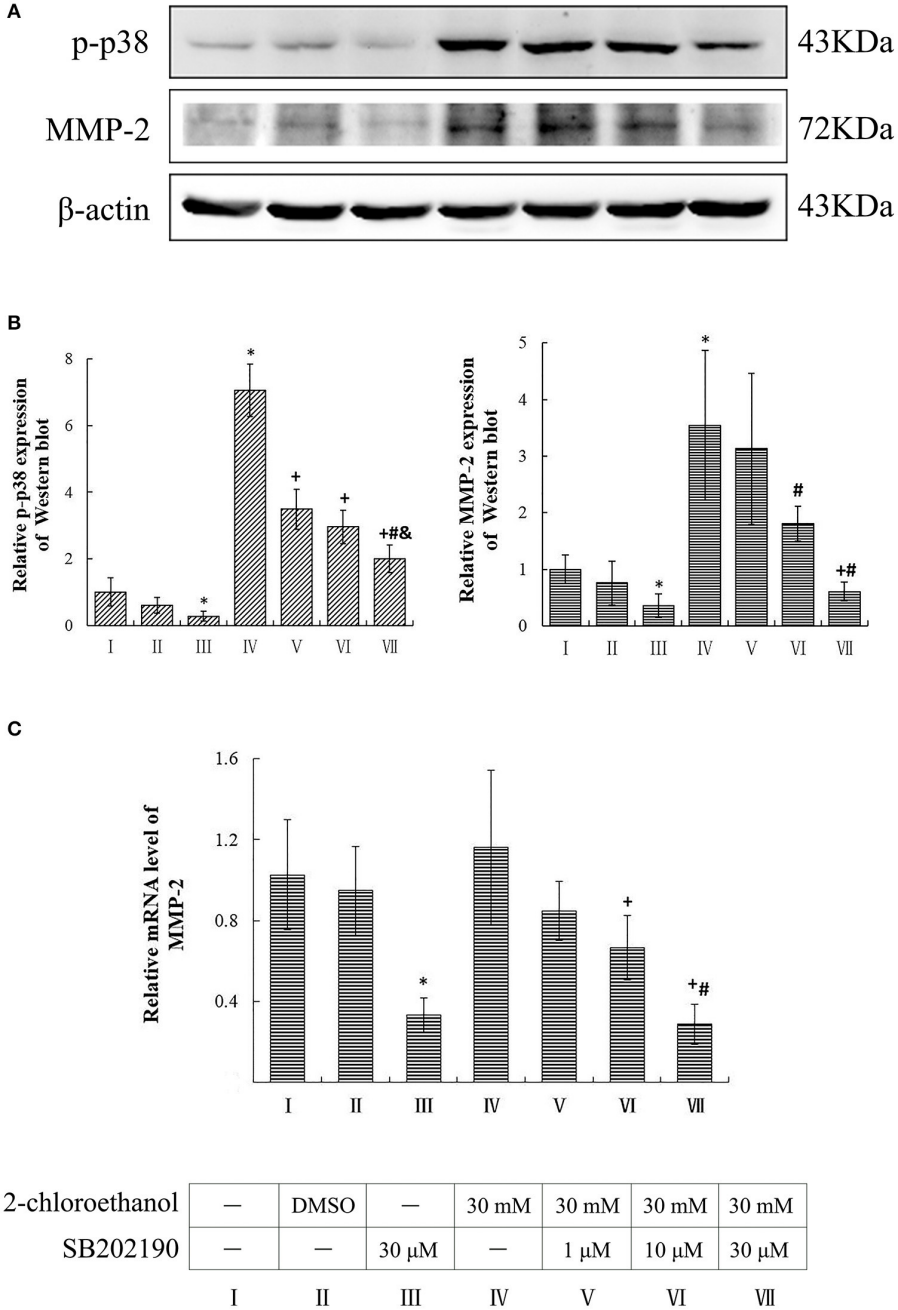
**Involvement of p38 in MMP-2 induction in primary cultured astrocytes exposed to 2-CE**. I to VII represent the blank control, solvent control, inhibitor control, exposure group, and low, middle and high dose of SB202190 inhibition group, respectively. **(A)** Western blot analysis. Images were the representative results of three separate experiments for each group. **(B)** Densitometric analysis of western blots. The relative intensity in arbitrary units was compared to β-actin and presented as fold change vs. the control group. **(C)** Quantitation of mRNA by real-time RT-PCR. The gene expression was normalized to GAPDH and presented as fold change vs. the control. Data expressed as mean ± SD were the results of three independent experiments from different litters of rats for each treatment, and analyzed by One-way ANOVA. Significant difference was defined as *p* < 0.05, and ^*^, vs. blank control; ^+^, vs. exposure group; ^#^, vs. low dose intervention group; ^&^, vs. middle dose intervention group.

Compared to the blank control group, the protein levels of p-p38 and MMP-2 in the inhibitor control group decreased significantly, whereas those in the exposure group increased significantly (*p* < 0.05, shown in Figures 3A,B). Moreover, the protein levels of p-p38 in SB202190 inhibition groups decreased significantly as compared to the exposure group, and those in the high dose of SB202190 inhibition group also decreased significantly compared to other intervention groups. Consistent with the changes of p-p38 protein levels, the MMP-2 protein levels in the middle and high dose of SB202190 inhibition groups decreased significantly compared to exposure group. In addition, the graph shown in Figure 3C disclosed the comparison of MMP-2 mRNA levels in the cells among groups, and indicated that the mRNA levels of MMP-2 in the inhibitor control group decreased significantly as compared to blank control group, and those in the middle and high dose of SB202190 inhibition groups also decreased significantly compared to the exposure group.

### Involvement of ERK in upregulation of MMP-2 expression in 2-CE treated astrocytes

**Figure 4:**
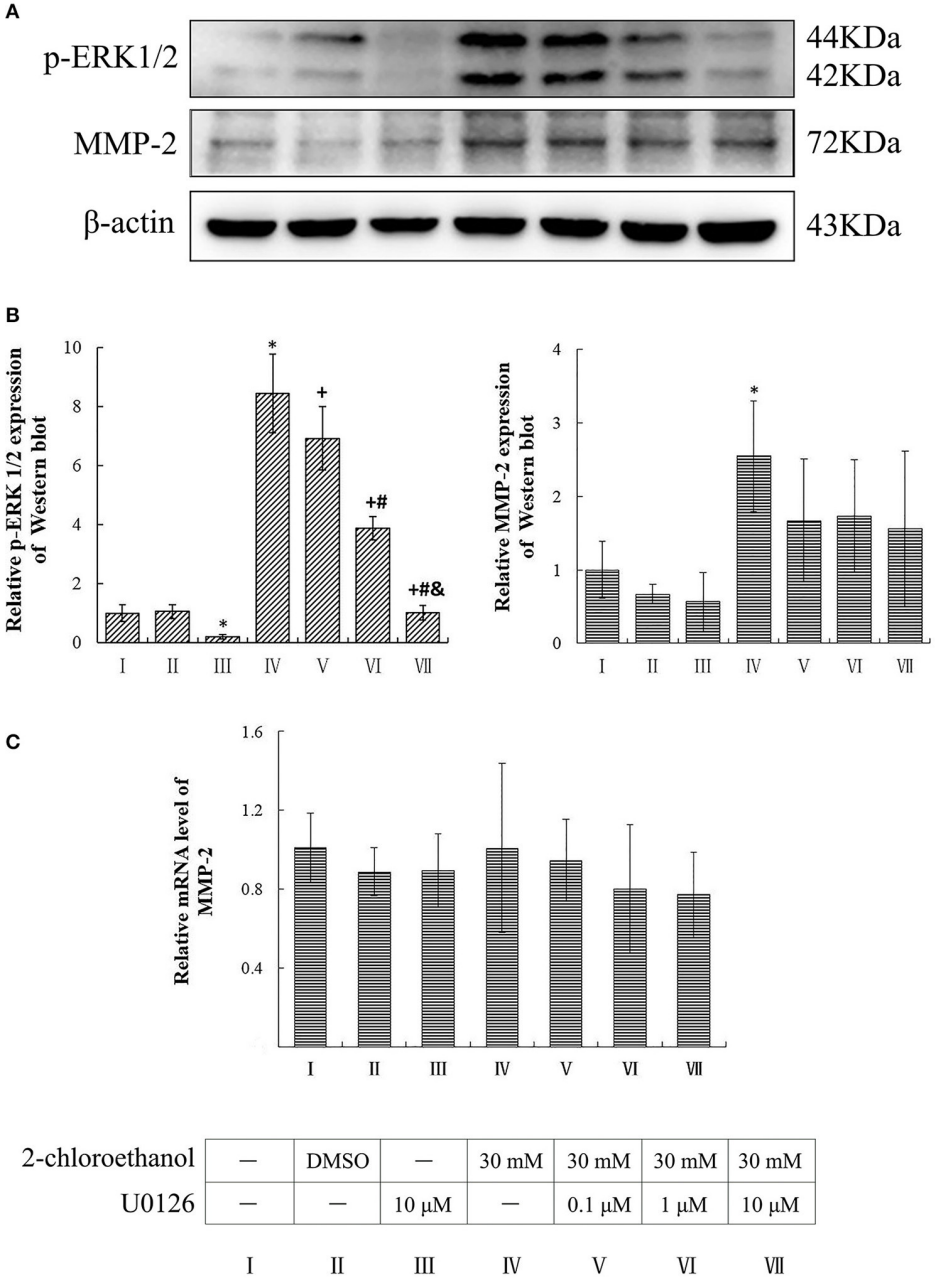
**Involvement of ERK in MMP-2 induction in primary cultured astrocytes exposed to 2-CE**. I to VII represent the blank control, solvent control, inhibitor control, exposure group, and low, middle and high dose of U0126 inhibition group, respectively. **(A)** Western blot analysis. Images were the representative results of three separate experiments for each group. **(B)** Densitometric analysis of western blots. The relative intensity in arbitrary units was compared to β-actin and presented as fold change vs. the control group. **(C)** Quantitation of mRNA by real-time RT-PCR. The gene expression was normalized to GAPDH and presented as fold change vs. the control. Data expressed as mean ± SD were the results of three independent experiments from different litters of rats for each treatment, and analyzed by One-way ANOVA. Significant difference was defined as *p* < 0.05, and ^*^, vs. blank control;^+^, vs. exposure group; ^#^, vs. low dose intervention group; ^&^, vs. middle dose intervention group.

Compared to the blank control group, the protein levels of p-ERK1/2 in the inhibitor control group decreased significantly, whereas those of p-ERK1/2 and MMP-2 in the exposure group increased significantly (*p* < 0.05, shown in Figures 4A,B). Moreover, the protein levels of p-ERK1/2 in the U0126 inhibition groups decreased significantly and dose dependently; however both protein and mRNA levels of MMP-2 in the intervention groups did not differ significantly as compared to the exposure group (shown in Figure 4C).

### Involvement of JNK in upregulation of MMP-2 expression in 2-CE treated astrocytes

**Figure 5:**
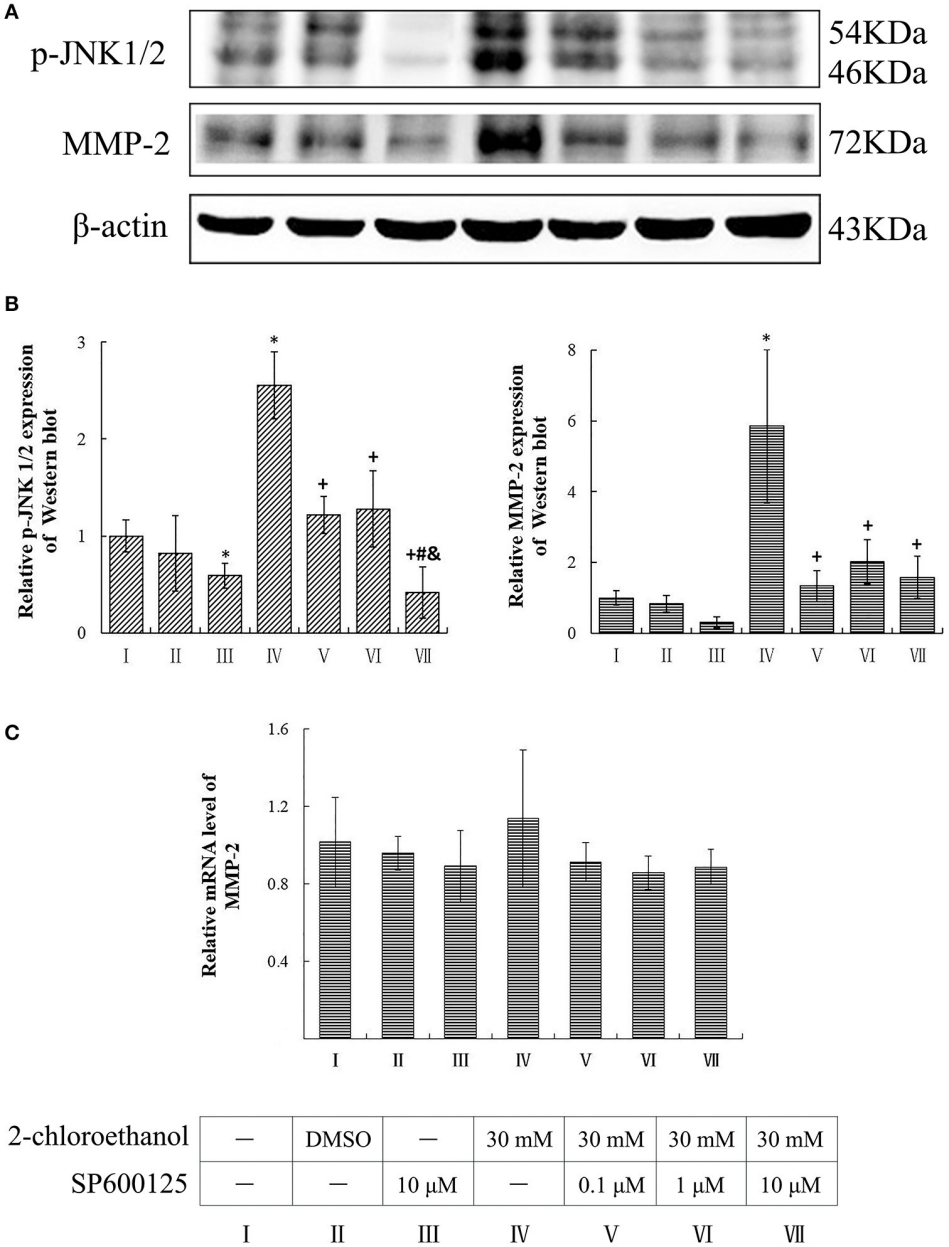
**Involvement of JNK in MMP-2 induction in primary cultured astrocytes exposed to 2-CE**. I to VII represent the blank control, solvent control, inhibitor control, exposure group, and low, middle and high dose of SP600125 inhibition group, respectively. **(A)** Western blot analysis. Images were the representative results of three separate experiments for each group. **(B)** Densitometric analysis of western blots. The relative intensity in arbitrary units was compared to β-actin and presented as fold change vs. the control group. **(C)** Quantitation of mRNA by real-time RT-PCR. The gene expression was normalized to GAPDH and presented as fold change vs. the control. Data expressed as mean ± SD were the results of three independent experiments from different litters of rats for each treatment, and analyzed by One-way ANOVA. Significant difference was defined as *p* < 0.05, and ^*^, vs. blank control;^+^, vs. exposure group; ^#^, vs. low dose intervention group; ^&^, vs. middle dose intervention group.

Compared to the blank control group, the protein levels of p-JNK1/2 in the inhibitor control group decreased significantly, whereas those of p-JNK1/2 and MMP-2 in the exposure group increased significantly (*p* < 0.05, shown in Figures 5A,B). Moreover, the protein levels of both p-JNK1/2 and MMP-2 in the intervention groups decreased significantly as compared to the exposure group, and those of p-JNK1/2 in the high dose of SP600125 inhibition group decreased significantly as compared to the other intervention groups. However, the protein levels of MMP-2 did not differ significantly among the intervention groups. On the other hand, the mRNA levels of MMP-2 did not differ significantly between the exposure group and intervention groups (shown in Figure 5C).

## Discussion

Results of present study suggested that the protein levels of MMP-2 in rat astrocytes could be upregulated by 2-CE treatment. The protein levels of MMP-2 in 30 mM 2-CE treated rat astrocytes increased 523%. Similarly, our previous *in vivo* study demonstrated that the protein level of MMP-2 in the brain increased markedly at the process of 1,2-DCE induced brain edema formation in mice (Wang et al., 2014). Collectively, these results suggested that upregulated MMP-2 expression by both 1,2-DCE and 2-CE occurred mainly at the translational level, including enhanced protein synthesis and decreased enzyme degradation. Since MMP-2 is constitutively expressed, and normally tethered to the cell surface as an inactive form after synthesis, it might be released from the cell membrane (Ogier et al., 2006). Accordingly, it is reasonable to assume that upregulated protein levels of MMP-2 in the brain of mice treated with 1,2-DCE might be partially resulted from direct effects of 2-CE generated from CYP2E1 mediated metabolism of 1,2-DCE *in vivo*.

Increasing evidences have suggested that astrocytes are the site of early dysfunction and damage induced by exogenous chemicals (Hazell, 2002). Astrocyte activation is manifested with an increase in proinflammatory cytokine production, and the release of neurotoxic factors such as MMPs (Hong et al., 2005; Rosenberg, 2009; Lew et al., 2013). Although the pathophysiological processes of brain edema are not completely understood, many reports have indicated that activation of astrocytes and the subsequent release of proinflammatory factors and neurotoxic factors might contribute to breakdown in BBB integrity (Cho et al., 2013; Malinsky et al., 2013). Therefore, generation of 2-CE *in vivo* might contribute to the formation of vasogenic cerebral edema in 1,2-DCE treated mice.

Furthermore, the molecular basis of the effects of 2-CE on modulation of MMP-2 in rat astrocytes has been assessed in present study, and the results clearly demonstrated that treatment of astrocytes with 2-CE could activate the MAPK signal pathway by enhancing the protein phosphorylation of p38, ERK1/2, and JNK1/2, which increased 705, 845, and 255%, respectively. By using the specific MAPK inhibitors, the relationship between upregulation of MMP-2 expression and activation of MAPK signal pathways was further investigated in 2-CE treated astrocytes. The protein levels of MMP-2 decreased markedly in response to suppressed protein levels of p-p38 and p-JNK1/2. However, the protein levels of MMP-2 in astrocytes did not change markedly in response to suppressed protein levels of p-ERK1/2. Thus, our results suggested that treatment of 2-CE could upregulate the protein levels of MMP-2 through activation of p-38 MAPK and JNK signal pathway. In another words, activation of MAPK signal pathways could play the key roles in upregulation of MMP-2 expression in 2-CE treated astrocytes.

The roles of MAPK signal pathways in modulation of MMP-2 expression have been testified by previous studies. It has been reported that the specific MAPK inhibitor effectively reduced the MMP-2 levels in rat cortical cultures after mechanical trauma injury (Wang et al., 2002). Additionally, study reported by Qin et al. (2015) disclosed that treatment with LPS decreased the mRNA and protein levels of occludin and ZO-1, and enhanced the phosphorylation of p38 MAPK and JNK, and expression of MMP-2 in the cerebral microvascular endothelial cells, which were attenuated by pretreatment with SB203580 or SP600125, which suggested that activation of p38 MAPK and JNK pathways were involved in the LPS-induced MMP-2 overexpression, and in turn contributed to disruption of blood brain barrier integrity. Thus, our results were consistent with their findings.

It has been well established that p38 was the first protein isolated from MAPK signal pathways, and plays the central roles in inflammation (Wu et al., 2004; Borders et al., 2008; Kant et al., 2011; Yang et al., 2014). It can be strongly activated by various stress conditions and inflammatory cytokines, and then migrates from the cytosol into nucleus or can transfer to the other parts of the cell, where it activates transcription factors or downstream kinases (Arthur and Ley, 2013). JNK has been shown to play the crucial roles in controlling cell proliferation and apoptosis. It is strongly activated in response to a variety of stress signals (Bas et al., 2015). The activation of JNK pathway could also result in gene transcription (Verma and Datta, 2012). ERKs in general, are pivotal regulators of proliferation and differentiation. They can be activated by many factors, and then translocate to the nucleus, where they activate various transcription factors, and ultimately leading to changes in gene expression (Wang et al., 2002; Piao et al., 2003; Fujimoto et al., 2007; Wu et al., 2010; Huang et al., 2013; Gladbach et al., 2014). Because the three kinds of MAPK signal pathways possess many similarities, they may undergo cross-talk at several levels. Until now, the accumulated evidence suggested that MMP-2 expression was regulated by the p38, ERK1/2 and JNK1/2 signal pathways in different cell types (Lee et al., 2014; Kim et al., 2015). However, to our knowledge this was the first report to demonstrate the molecular mechanisms underlying MMP-2 upregulation in 2-CE treated rat astrocytes.

## Author contributions

QS designed, performed, and interpreted the experiments and wrote the manuscript. TW performed parts of biochemistry experiments and edited the manuscript. HT, YL, FZ, and GW edited the manuscript. YJ conceived the study, designed and interpreted experiments and revised the manuscript.

### Conflict of interest statement

The authors declare that the research was conducted in the absence of any commercial or financial relationships that could be construed as a potential conflict of interest.
